# Trajectory-Ordered Objectives for Self-Supervised Representation Learning of Temporal Healthcare Data Using Transformers: Model Development and Evaluation Study

**DOI:** 10.2196/68138

**Published:** 2025-06-04

**Authors:** Ali Amirahmadi, Farzaneh Etminani, Jonas Björk, Olle Melander, Mattias Ohlsson

**Affiliations:** 1 Center for Applied Intelligent Systems Research in Health Halmstad University Halmstad Sweden; 2 Center for Applied Intelligent Systems Research Halmstad University Halmstad Sweden; 3 Department of research and development (FoU) Region Halland HALMSTAD Sweden; 4 Division of Occupational and Environmental Medicine Lund University Lund Sweden; 5 Department of Clinical Sciences Lund University Lund Sweden; 6 Centre for Environmental and Climate Science Lund University Lund Sweden

**Keywords:** patient trajectories, disease prediction, representation learning, masked language mode, deep learning, BERT, electronic health record, language mode, transformer, heart failure, alzheimer disease, prolonged health of stay, effectiveness, temporal

## Abstract

**Background:**

The growing availability of electronic health records (EHRs) presents an opportunity to enhance patient care by uncovering hidden health risks and improving informed decisions through advanced deep learning methods. However, modeling EHR sequential data, that is, patient trajectories, is challenging due to the evolving relationships between diagnoses and treatments over time. Significant progress has been achieved using transformers and self-supervised learning. While BERT-inspired models using masked language modeling (MLM) capture EHR context, they often struggle with the complex temporal dynamics of disease progression and interventions.

**Objective:**

This study aims to improve the modeling of EHR sequences by addressing the limitations of traditional transformer-based approaches in capturing complex temporal dependencies.

**Methods:**

We introduce Trajectory Order Objective BERT (Bidirectional Encoder Representations from Transformers; TOO-BERT), a transformer-based model that advances the MLM pretraining approach by integrating a novel TOO to better learn the complex sequential dependencies between medical events. TOO-Bert enhanced the learned context by MLM by pretraining the model to distinguish ordered sequences of medical codes from permuted ones in a patient trajectory. The TOO is enhanced by a conditional selection process that focus on medical codes or visits that frequently occur together, to further improve contextual understanding and strengthen temporal awareness. We evaluate TOO-BERT on 2 extensive EHR datasets, MIMIC-IV hospitalization records and the Malmo Diet and Cancer Cohort (MDC)—comprising approximately 10 and 8 million medical codes, respectively. TOO-BERT is compared against conventional machine learning methods, a transformer trained from scratch, and a transformer pretrained on MLM in predicting heart failure (HF), Alzheimer disease (AD), and prolonged length of stay (PLS).

**Results:**

TOO-BERT outperformed conventional machine learning methods and transformer-based approaches in HF, AD, and PLS prediction across both datasets. In the MDC dataset, TOO-BERT improved HF and AD prediction, increasing area under the receiver operating characteristic curve (AUC) scores from 67.7 and 69.5 with the MLM-pretrained Transformer to 73.9 and 71.9, respectively. In the MIMIC-IV dataset, TOO-BERT enhanced HF and PLS prediction, raising AUC scores from 86.2 and 60.2 with the MLM-pretrained Transformer to 89.8 and 60.4, respectively. Notably, TOO-BERT demonstrated strong performance in HF prediction even with limited fine-tuning data, achieving AUC scores of 0.877 and 0.823, compared to 0.839 and 0.799 for the MLM-pretrained Transformer, when fine-tuned on only 50% (442/884) and 20% (176/884) of the training data, respectively.

**Conclusions:**

These findings demonstrate the effectiveness of integrating temporal ordering objectives into MLM-pretrained models, enabling deeper insights into the complex temporal relationships inherent in EHR data. Attention analysis further highlights TOO-BERT’s capability to capture and represent sophisticated structural patterns within patient trajectories, offering a more nuanced understanding of disease progression.

## Introduction

In modern health care, electronic health records (EHRs) are crucial as comprehensive repositories encompassing a wide range of patient data, including diagnoses, medications, treatments, laboratory data, and demographic information. The accumulation of EHR data longitudinally builds EHR trajectories, sometimes called patient trajectories. This information serves as an important resource for assessing a patient’s current health status and predicting potential health risks. Using advanced deep learning (DL) models with this extensive data opens the possibility of making predictions, such as disease risks, treatment outcomes, and patient prognoses. This possibility equips health care providers with the tools to make informed decisions, ultimately improving patient care, optimizing interventions, and reducing health care costs.

However, developing DL methods for modeling EHR data is full of challenges. Effectively addressing the complexity of the heterogeneous data extracted from patients’ EHR [1-4], capturing short- and long-term relationships between medical codes across various visits, contending with the scarcity of publicly available EHR sources, and navigating the vast diversity of diseases pose significant hurdles. In addition, ensuring transparency and explainability in the predictions made by DL techniques demands substantial effort [5-7].

Current state-of-the-art models for EHR trajectory data are based on transformer architecture [8], in particular, models inspired by the Bidirectional Encoder Representations from Transformers (BERT) [9] architecture [10-14]. Such models aim to capture short- and long-term relationships through a task-agnostic representation learning (RL) approach, where the masked language model (MLM) pretraining objective is very common.

The BERT-inspired models for EHR trajectory data can be examined from various perspectives: data and model size, used data modalities, architecture, and pretraining objectives. Within this context, we focus on the primary and auxiliary pretraining objective functions designed to enhance the capabilities of the learned representation.

The primary objective typically takes the form of a generative task, benefiting from its enhanced ability to grasp intricate relationships. MLM objectives have found widespread application in EHR trajectory prediction tasks, largely owing to the capabilities of BERT models to learn the context (both past and future simultaneously) [10,11,15-22]. The autoregressive pretraining objective [23,24] serves as the other approach for RL of EHR trajectory data. It accomplishes this by predicting upcoming medical events, such as the codes for the next day or the subsequent visit, leveraging the patient’s historical data [25-27].

A range of auxiliary pretraining objectives has been proposed to enhance the RL performance, incorporating either external knowledge or using contrastive learning. Examples of the former include Shang et al [15] and Amirahmadi et al [28] that predicted medications based on diagnoses and diagnoses based on medication to induce relationships within the diagnoses and interventions in the learned representation. Med-BERT [11] introduced a length of stay auxiliary prediction task to enrich contextual information about the severity of patients’ health conditions. CEHRT-BERT [16] and Claim-PT [27] incorporated visit type predictions (eg, inpatient and outpatient visits) to represent external domain knowledge into the model, mitigating the effect of sparse codes based on the observation that different medical concepts are associated with different visit types.

RareBERT [19] introduced a 1-class classification objective to improve model performance for rare disease prediction. Similarly, AdaDiag [18] added a domain classifier to distinguish data from different institutes and enhance the generalizability and robustness of the learned representation against dataset shifts.

From the contrastive learning category, we find Hierarchical BEHRT (Hi-BEHRT) [17] that used bootstrap your own latent (BYOL) [29] similarity learning, operating under the assumption that varying augmentations of the same input yield similar representations, thereby enhancing the latent representation of the network. Rapt [21] trained the transformer to differentiate between different patient trajectories, relying on the Euclidean distance between their last visits to enrich the RL’s understanding of their health condition. In addition, Rapt used another auxiliary objective, like the next sentence prediction, to discern whether a trajectory belongs to a specific patient or constitutes a fusion of various patient trajectories, facilitating the learning of trends within health trajectories. Generative Adversarial Networks Enhanced Pretraining (GRACE) [22], addressing the EHR data insufficiency challenge, incorporated a real or fake contrastive learning objective to distinguish authentic EHR data from generative adversarial network (GAN)–generated EHR data within the MLM framework.

Instances of medical events can influence the likelihood of other medical events, shaping the trajectory of patients toward more or less severe health conditions. Moreover, numerous medical events exhibit semicausal relationships through chains of probability paths that have not been extensively studied [30-33]. The relative intervals between medical events play a pivotal role in adjusting a patient’s trajectory and are a key factor for the RL model. In pretrained language models, similar concepts are applied to comprehend the global coherence of data. BERT [9] used a next-sentence prediction (NSP) to capture the global relations between sentences. However, Liu et al [34] showed that NSP does not generate a positive impact, and Lan et al [35] speculate that the reason lies in the simplicity of NSP and its overlap with the MLM loss. Consequently, they replaced NSP with sentence order prediction, prioritizing coherence prediction over topic prediction [36,37]. Before the mentioned studies, researchers improved their machine translation, constituency parsing models, and object detection by altering the order of input [38-40]. Vinyals et al [41] delved into the problem that, while in theory incorporating the order of sequences should not have a significant impact when using complex encoders due to their nature as universal approximators, in practice, it does matter due to underlying nonconvex optimization and more fitting priors.

Furthermore, EHR trajectories encompass a history of abnormal health conditions, including diseases, laboratory data, and prescribed interventions like medications and procedures. The occurrence of certain diseases can alter a person’s health trajectory and increase the probability of other illnesses. Similarly, interventions often mitigate the severity of conditions at the cost of raising other health risks. Thus, every medical event, whether it involves diseases or medications, can serve as a cause, complication, or early symptom of the recorded codes [42]. This study will demonstrate that order objectives, besides the context, enhance the model performance by learning more structural information. In summary, the contributions are mentioned in Textbox 1.

**Overview of the study's main contributions.**

- We have examined the ability of Bidirectional Encoder Representations from Transformers–inspired models to capture the representation of sequential information of medical codes. Our findings indicate that although transformers and language models excel at identifying global dependencies based on contextual information, learning the order of diseases and medications can be challenging, especially for patients with long trajectories.
- We introduced a novel “trajectory order objective” self-supervised auxiliary task to the masked language model (MLM). This new objective was applied at both the single code and visit levels, and we demonstrated its efficacy in enhancing the original MLM by evaluating it on heart failure, Alzheimer disease, and prolonged length of stay downstream prediction tasks on 2 distinct datasets.
- We introduced the conditional code swapping and conditional visit swapping functions built on the “conditional-based order of medical codes.” This function allows swapping more frequent consecutive repetitions, enabling the model to systematically learn the patterns of transitions at both the single code and visit levels.
- We demonstrated how adding the new objective reshapes the attention behavior of the transformer model and encourages the model to attend to relations between 2 sets rather than 2 individual codes, enabling the learning of more complex structural relationships.

## Methods

### Data

In this study, we extracted medical diagnoses and medication histories from 2 distinct (EHR) trajectory datasets, namely the Medical Information Mart for Intensive Care IV (MIMIC-IV) hosp module [43] and the Malmo Diet and Cancer Cohort (MDC) [44]. These 2 datasets have unique characteristics that suit our research objectives.

The MIMIC-IV hosp module encompasses a rich, detailed collection of inpatient EHR trajectories, comprising a total of approximately 173,000 patient records recorded during 407,000 visits. The data spans from 2008 to 2019, offering a comprehensive view of patient journeys within the hospital setting. MIMIC-IV hosp module contains approximately 10.6 million medical codes associated with a large volume of patients. The MDC data is a prospective cohort from Sweden. It consists of approximately 30,000 individuals residing in the municipality of Malmo (southern Sweden) between 1991 and 1996. The cohort was recruited from a total population of about 74,000 individuals, encompassing all men born between 1923 and 1945 and all women born between 1923 and 1950. All inpatient and outpatient visits between 1992 and 2020 have been recorded, resulting in a total of 531,000 visits. Although the MDC dataset has fewer overall samples, it excels in providing a more extensive patient history, averaging 257 codes per patient compared to MIMIC-IV’s 61 (for more details, refer to Multimedia Appendix 1).

Diseases and medications in both datasets are classified using the *ICD* (*International Classification of Diseases and Related Health Problems*) and Anatomical Therapeutic Chemical Code (ATC), respectively. These coding systems follow a hierarchical format, providing granular details about diseases or medications based on code length.

To facilitate our self-supervised pre-training, supervised fine-tuning, and final testing, we randomly partitioned the extracted cohort into 3 subsets: 70%, 20%, and 10%, respectively. For further details on the specifications of the MIMIC-IV and MDC datasets, refer to Tables 1 and 2.

**Table 1:** Summary statistics of the Medical Information Mart for Intensive Care IV (MIMIC-IV) dataset.

	Pretraining dataset	Fine-tuning dataset	Test dataset	Total dataset
Patients, n	121,000	36,000	16,000	173,000
Visits, n	285,000	86,000	37,000	408,000
*ICD-9*^a^ codes, n	1,940,000	579,000	248,000	2,767,000
ATC^b^ codes, n	5,511,000	1,655,000	688,000	7,854,000
All codes, n	7,451,000	2,234,000	937,000	10,622,000

^a^ICD-9: International Classification of Diseases, Ninth Revision.

^b^ATC: Anatomical Therapeutic Chemical Code.

**Table 2:** Summary statistics of the Malmo Diet and Cancer Cohort (MDC) dataset.

	Pretraining dataset	Fine-tuning dataset	Test dataset	Total dataset
Patients, n	21,000	6000	3000	30,000
Visits, n	373,000	107,000	52,000	531,000
*ICD-10*^a^ codes, n	1,155,000	331,000	161,000	1,647,000
ATC^b^ codes, n	4,185,000	1,223,000	580,000	5,988,000
All codes, n	5,339,000	1,554,000	741,000	7,634,000

^a^ICD-10: International Statistical Classification of Diseases, Tenth Revision.

^b^ATC: Anatomical Therapeutic Chemical Code.

### Ethical Considerations

The use of the MDC dataset for this study was approved by the Ethics Review Board of Sweden (Dnr 2023-00503-01). Regarding the MIMIC-IV dataset, all protected health information (PHI) is officially deidentified. It means that the deletion of PHI from structured data sources (eg, database fields that provide age, genotypic information, and past and current diagnosis and treatment categories) is performed in compliance with the HIPAA (Health Insurance Portability and Accountability Act) standards in order to facilitate public access to the datasets.

### Data Processing and Problem Formulation

Each dataset D comprises a set of patients P, D = {P^1^,P^2^,...,P^|D|^}. In our study, we considered a total of |D|= 172,980 patients for MIMIC-IV and |D|= 29,664 patients for the MDC cohort. We represent each patient’s longitudinal medical trajectory through a structured set of visit encounters. Given the continuous recording of medical codes in the MDC cohort, we define a visit entity V for each code and all previous subsequent codes occurring within a 6-month time window. For the MIMIC-IV, we used the predefined visits. This representation is denoted as P, P^i^ = {V^i^_1_, V^i^_2_, …, V^i^_O_}, where O represents the total number of visit encounters for patient I. For each visit, V_j_^i^ = I_j_ ∪M_j_ is the union of all diagnoses codes I_j_ ⊂ I and prescribed medications M_j_ ⊂ M that are recorded for the P^i^ at visit V_j_^i^. To reduce the sparsity, we excluded less frequently occurring medical codes and retained only the initial 4 digits of *ICD* and ATC codes (refer to Multimedia Appendix 1). This process resulted in 2195 *ICD-9* (*International Classification of Diseases, Ninth Revision*) and 137 ATC-5 unique codes for the MIMIC-IV dataset and 1558 *ICD-10* (*International Statistical Classification of Diseases, Tenth Revision*) and 111 ATC-5 unique codes for the MDC dataset. In addition, for the MIMIC-IV, we converted medication data from National Drug Code (NDC format to ATC format to benefit from its hierarchical structure and improve comparability.

To guide the model in understanding changes in encounter times and the structure of each patient’s trajectory, like BERT, we used special tokens. A CLS token is placed at the beginning of each patient's trajectory, while a SEP token is inserted between visits. Consequently, each patient trajectory is represented as *P^i^={CLS, V_1_^i^,SEP, V_2_^i^,SEP,…,V_0_^i^,SEP},* providing the model with valuable context for analysis and prediction.

### Heart Failure Prediction

The primary downstream task is heart failure (HF) prediction, where the model predicts the incidence of the first HF *ICD* codes I_N=HF_ on the Nth visit, given the patient’s previous history of diagnosis and medication intervention:



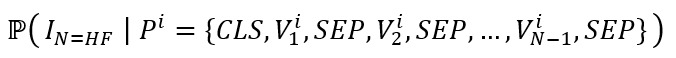



For each patient’s trajectory, if there were no occurrences of the target disease, it is considered a negative case; otherwise, we excluded the first visit with target codes and all subsequent visits and considered it a positive case. Furthermore, all ATC codes related to HF treatment are excluded. To mitigate trajectory length bias, trajectories with fewer than 10 and 30 visits are excluded from MIMIC IV and the MDC dataset, respectively, ensuring an equal average number of visits within positive and negative cases. Following these preprocessing steps, we obtained Cohorts with a history of 5 visits for MIMIC-IV and 20 visits for the MDC dataset on average. As a remark, in the MDC Cohort, this task is equivalent to predicting HF in the next 6 months, as per the preprocessing design.

### Alzheimer Disease Prediction

The next downstream task is Alzheimer disease (AD) prediction. Similar to the HF prediction downstream task, the model predicts the occurrence of the first AD *ICD* codes I_N=AD_ on the Nth visit, considering the patient’s previous history of diagnoses and medication interventions:



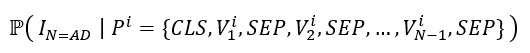



We followed the HF prediction preprocessing steps. The AD downstream prediction task was tested on the MDC dataset.

### Prolonged Length of Stay Prediction

To explore the adaptability of pretrained models to a distinct task from the one they were initially trained on (code prediction), we used the prediction of prolonged length of stay (PLS) as a binary classification downstream task. In this task, the model is assigned the objective of predicting the PLS in the Nth visit based on a patient’s diagnoses and medications in previous visits in the MIMIC-IV dataset:



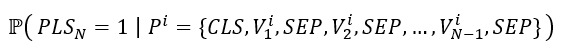



Consistent with [45], patients with a stay longer than 5 days were considered positive cases for PLS. To maintain consistency with the HF prediction task, trajectories with fewer than 3 visits were excluded, and the average number of visits was equalized between positive and negative cases. Following these steps, we obtained a Cohort with an average history of 5 visits (refer to Multimedia Appendix 1).

## Methods

Recent studies have underscored the effectiveness of using multihead transformer architecture and MLM self-supervised learning in the domain of EHR trajectory modeling. While these methods have exhibited superior performance in various contexts, we focus on investigating their limitations related to sequential order learning and propose enhancements to address this issue.

A fundamental aspect of EHR trajectory modeling is the critical role of the sequential order of medical events in guiding patients’ trajectories. For instance, the timely administration of appropriate medical interventions can significantly alter a patient’s trajectory, either improving the severity of their condition or, conversely, leading to unintended side effects.

### Models Objective

Our approach involves pretraining a transformer model on 2 distinct generative and contrastive self-supervised learning objectives: MLM and Trajectory-Order Objective (TOO). The MLM is crafted to learn the context, while TOO is designed to capture relations between local contexts. By simultaneously training the model on both of these objectives, we aim to leverage the entire set of patient trajectories and acquire a more comprehensive data representation.

#### MLM

The MLM generative task is used to learn the contextual dependencies among medical codes. In this paper, we corrupt the input by randomly masking the medical codes in each patient’s trajectory, denoted as *P^i^*_corrupted_, and train the model to maximize the likelihood of the masked codes, denoted as x^k^:



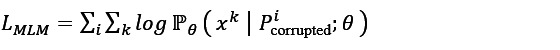



where is the conditional probability modeled by a deep neural network with parameter . We used a sliding window approach across patients’ trajectories to generate additional samples for the MLM objective (refer to Multimedia Appendix 1).

In the trajectory order objective, we train the transformer model to learn the relative positions of local features across 2 hierarchical levels, visits and medical codes. The TOO task helps the model gain insights into both causal and noncausal relationships within medical codes and visits. We achieved this by permuting each patient’s trajectory, using them as negative samples, while the unpermuted sequences served as positive examples for the TOO self-supervised contrastive learning task.



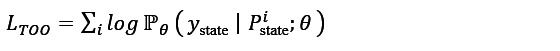



**Figure 1:**
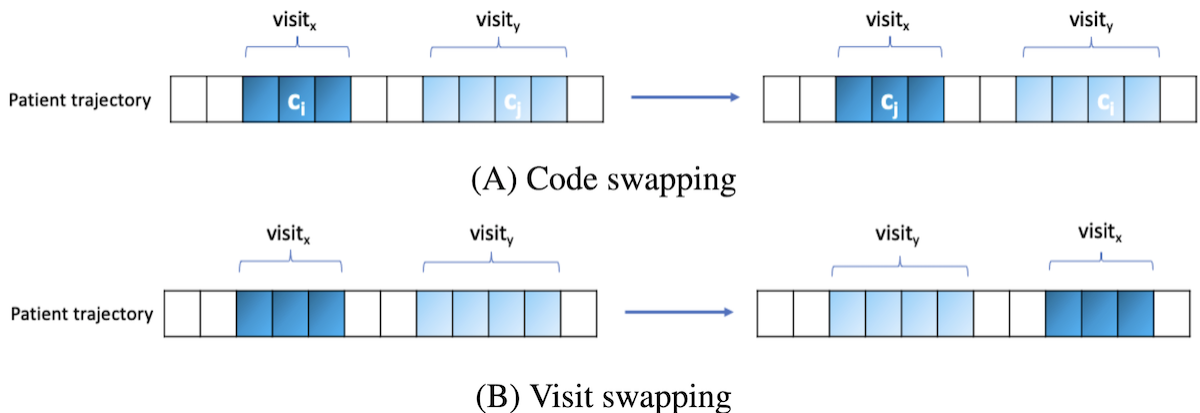
Code versus visits swapping. (A) Code swapping does not alter the visit structures of patient trajectories and only substitutes one medical code with another medical code in a different visit. (B) Visits swapping substitutes one visit, along with all its contents, with another visit, further disrupting the relative-time-wise dependencies between diagnoses and medications.

In this equation, state ∈ {permutated, ordered} denotes whether the trajectory *P*^i^ is ordered or permutated, and y_state_ ∈{0,1} is the corresponding label. We implemented the TOO task at the code level and visit level, by code swapping and visit swapping, respectively (refer to Figure 1).

Code swapping: We initiated code swapping by swapping codes between different visits of a patient’s trajectory. Given the complexity, especially for long trajectories, we designed and implemented 2 distinct methods. First, we randomly selected and swapped a subset of code pairs c_i_ and c_j_ with uniform probability, called random code swapping (RCS).

Second, to further facilitate the learning process for the transformer model and guide it toward more meaningful patterns, we introduced a conditional code swapping function (CCS; c_i_, c_j_). The idea is to prioritize code pairs that show a temporal dependency. The CCS function will provide a numerical estimate for such temporal relations, and in practice, code pairs (c_i_, c_j_) with large CCS (c_i_, c_j_) values are sampled more often than pairs with smaller values. We defined the CCS function as follows:



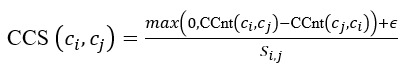



**Figure 2:**
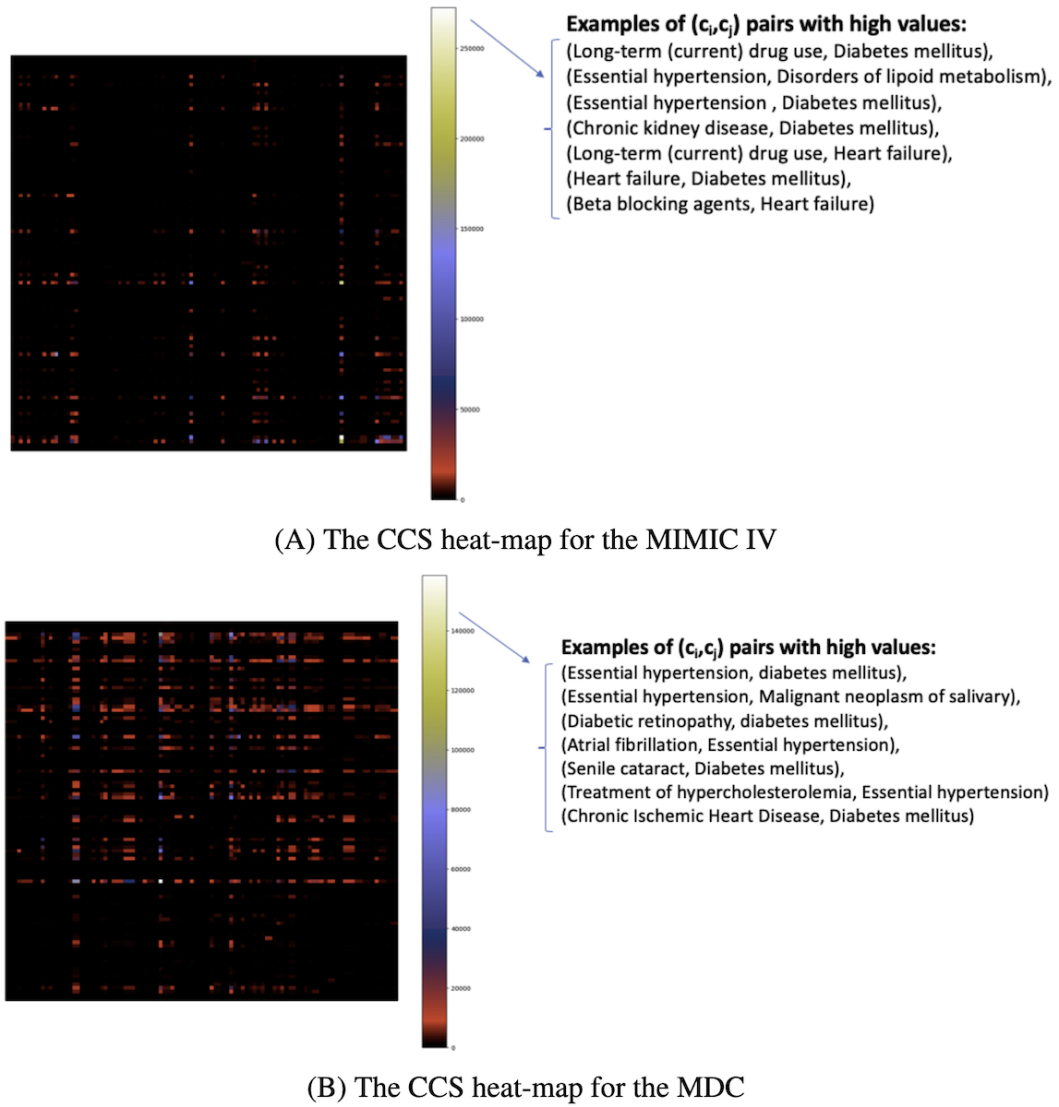
The conditional code swapping matrix heat map for a subset of medical codes in the medical information mart for Intensive Care IV and Malmo Diet cohort datasets.

Here, CCnt(c_i_, c_j_) is the count of all occurrences of code pairs (c_i_, c_j_) with the condition that code c_i_ appears after code c_j_ and that they are located in different visits. The count is performed over all patient trajectories in the pretraining dataset. The max operator in the nominator forces the CCS function to only consider the simple temporal dependencies that code c_i_ follows code c_j_. In other words, the maximum operator transforms the bidirectional transition graph between 2 medical codes into a unidirectional graph based on observations in the pretraining dataset. To account for a possible difference in the number of diagnoses and the number of medications, the CCS function was adjusted with a scaling factor S_i,j_ (refer to Multimedia Appendix 1, for more details). Finally, ε is a small number added to allow for a nonzero probability of selecting code pairs regardless of the relation between CCnt(c_i_, c_j_) and CCnt(c_j_, c_i_). Figure 2 show the CCS values, as a heatmap, for a selection of code pairs (c_i_, c_j_) for both datasets used in this study.

**Figure 3:**
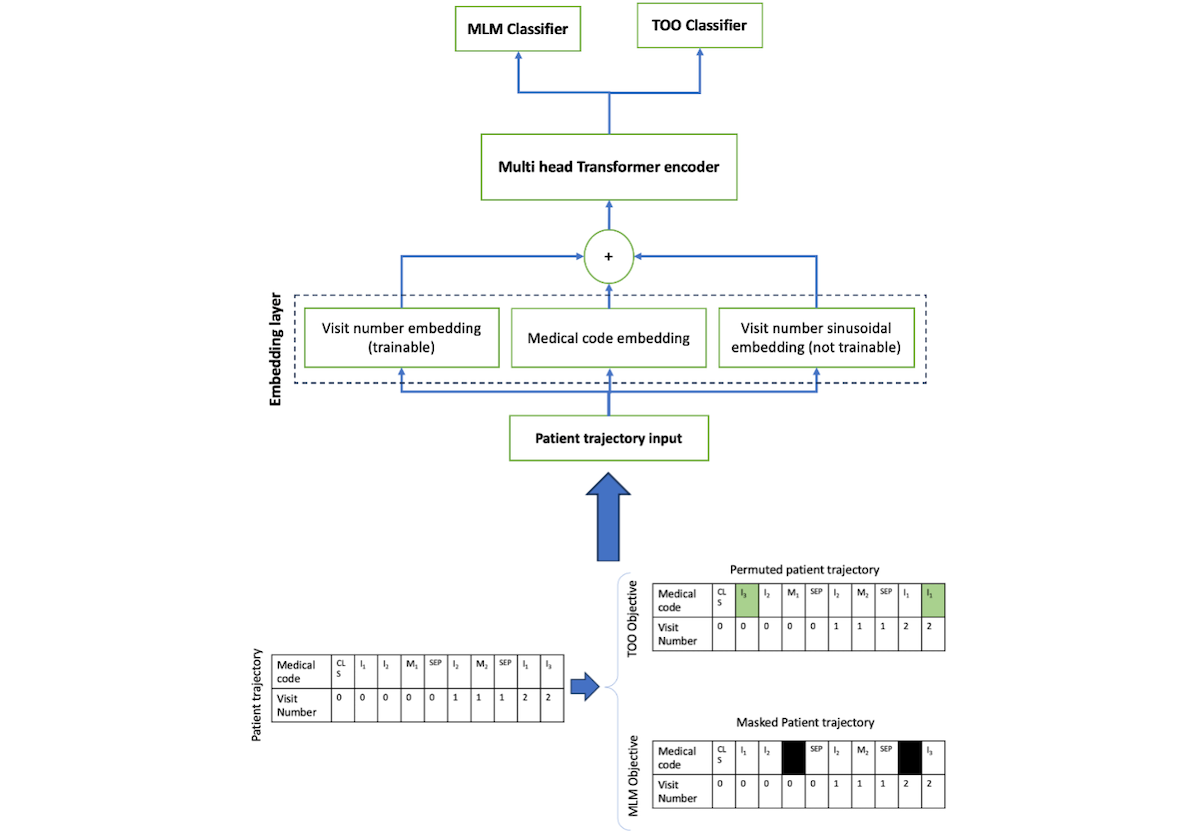
Trajectory order objective-Bidirectional Encoder Representations from Transformers architecture and example patient trajectory input. MLM: masked language modeling; TOO: trajectory-order objective.

#### Visits Swapping

Visit swapping aims to teach the model the coherence between different levels of local features in the global context. Instead of swapping positions of individual c_i_ and c_j_ codes, this method involves swapping the positions of all medical codes in visit x with those in visit y. Similar to code swapping, we implemented 2 methods for swapping visits. In the first method, we randomly sampled 2 visits x and y, with a uniform probability, and swapped them, denoted random visit swapping (RVS).

Second, we introduced the conditional visit swapping (CVS) function to prioritize among the visits to swap. This prioritization is based on the presence of codes within the visits that exhibited the simple temporal relation from the CCS approach as expressed by the CCS function above. To that end, the CVS function is calculated as the sum of CCS scores for all medical codes within visits x and y:



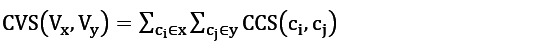



Similar to the way the CCS function was used for code swapping, pairs of visits with large CVS(v_x_,v_y_) values are sampled more often than visit pairs with lower values.

Figure 2 shows the CCS matrix heat map for a subset of medical codes in the MIMIC-IV and MDC datasets. Each row and column represents a medical code, and the heat map indicates the CCS score for all combinations of medical codes. Pairs with higher scores (indicated by lighter colors here) are more likely to be swapped with each other. Due to the long-tail distribution of medical codes and the predominance of less frequent codes, only a subset of the more frequent codes is displayed here. Examples of medical code pairs with the highest CCS scores are printed on the right side. For instance, in the MIMIC-IV dataset, “chronic kidney disease” and “diabetes mellitus” exhibit one of the highest CCS scores, suggesting that kidney disease frequently follows diabetes (but not vice versa). Similarly, in the MDC dataset, “atrial fibrillation” and “essential hypertension” have a high CCS score, indicating that atrial fibrillation often appears after hypertension. Training the model explicitly on such relationships allows it to learn more relevant connections between diseases and medications.

### Model Architecture

In this study, we used a multihead attention transformer encoder, drawing inspiration from BERT [8,9]. The model architecture, illustrated in Figure 3, includes an embedding module, a multihead attention transformer encoder, a feed-forward layer, and 2 classifier heads. The embedding module integrates medical codes with their associated temporal information. To capture the temporal dynamics, each medical code is paired with a visit sequence number, represented by 2 types of embeddings: a trainable visit number embedding and a nontrainable sinusoidal embedding, added together. These embeddings are summed with the medical code embeddings to form the input tensor X_0_, which is then passed through a standard transformer encoder to obtain the transformed representation X_1_:



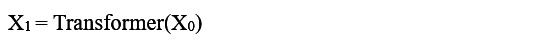



The learned representation is then passed to both the MLM and TOO classifier heads. The MLM classifier is a large softmax applied along the vocabulary dimension, used for predicting masked tokens. The TOO classifier is a binary classifier responsible for predicting the correct temporal order of the medical codes.

### Pretraining TOO-BERT

In the pretraining phase, we adopted a multitask learning approach to train the transformer network on both the MLM and TOO tasks simultaneously. For each batch, we alternated between these 2 objectives and performed gradient descent-based optimization on the weighted sum of their respective losses. This strategy allowed the model to learn both tasks cohesively, mitigating the risk of catastrophic forgetting that could occur if the objectives were trained sequentially [46,47] (ie, training on MLM first and then on TOO).

The total loss function for pretraining was defined as follows.

Each patient trajectory consists of a sequence of diagnoses, medications, and their associated visit sequence numbers, which are processed as input to TOO-BERT. The model includes an embedding layer, a multihead transformer encoder, and 2 classifier heads. First, all medical codes and their temporal information are embedded in the embedding layer. The combined embeddings are then passed through the multihead transformer encoder, followed by the MLM and TOO classifiers.



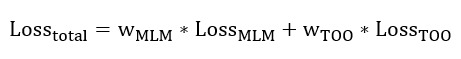



where W_MLM_ and W_TOO_ represent the weights assigned to each loss.

For the MLM objective, we randomly masked 15% [9] of the medical codes (equation 4). Similar to BERT and Med-BERT, during the masking process, each code had an 80% (n/N) chance of being replaced by [Mask], 10% (n/N) by a random code, and 10% (n/N) remained unchanged.

In the TOO task, we trained the model to classify the permuted trajectories using RCS, CCS, RVS, and CVS methods. We initially evaluated the model’s capability on the TOO task across various permutations.

### Fine-Tuning for Downstream Task

Following the pretraining phases for trajectory representation learning, we added a Bidirectional Gated Recurrent Unit (Bi-GRU) classifier head on top of the pretrained network, similar to Med-BERT, and finetuned it using the fine-tuning split for each specific downstream task. To enhance the fine-tuning process, we incorporated a layer-wise learning rate decay strategy [48,49] with gradient descent to decrease the weight changes in the initial layers compared with the later layers, thereby retaining the basic knowledge acquired during pretraining. In the final step, we compared the performance of our models against Logistic Regression, Random Forest, MLP, Bi-GRU, and a pretrained transformer with only the MLM objective.

### Implementation Details

We set aside 10% (12000/121000 in the MIMIC-IV and 2000/21000 in the MDC dataset) of the pretraining dataset for monitoring the transformer’s performance on the MLM and TOO pretraining objectives. The fine-tuning dataset was divided into 5 splits. For each iteration, we fine-tuned the pretrained models and trained the baseline models on 4 splits, using the remaining portion for early stopping. The reported results represent the average and SD of the performance across the 5 trained models on the isolated test dataset.

For the pretraining phase, we used a neural network featuring 5 self-attention heads and one transformer encoder with a d_k_ = d_v_ = d_x_ = 36 (refer to Multimedia Appendix 1), comprising approximately 300,000 learnable parameters. In each dataset, we computed the length of the trajectory for all patients and considered the 0.7 quantiles of the trajectory lengths as the maximum sequence length. For trajectories exceeding this length, we applied a moving window to generate additional augmented pretraining samples. We used the Adam optimizer with a learning rate of 7e-5, a weight decay of 0.015, and a dropout rate of 0.1, and trained the model until the loss curve stabilized. The MLP network comprises 2 hidden layers with 250 and 100 nodes, and the Bi-GRU network features one bidirectional GRU layer with 64 hidden nodes.

## Results

### Evaluation of Pretraining on the TOO Auxiliary Task

**Figure 4:**
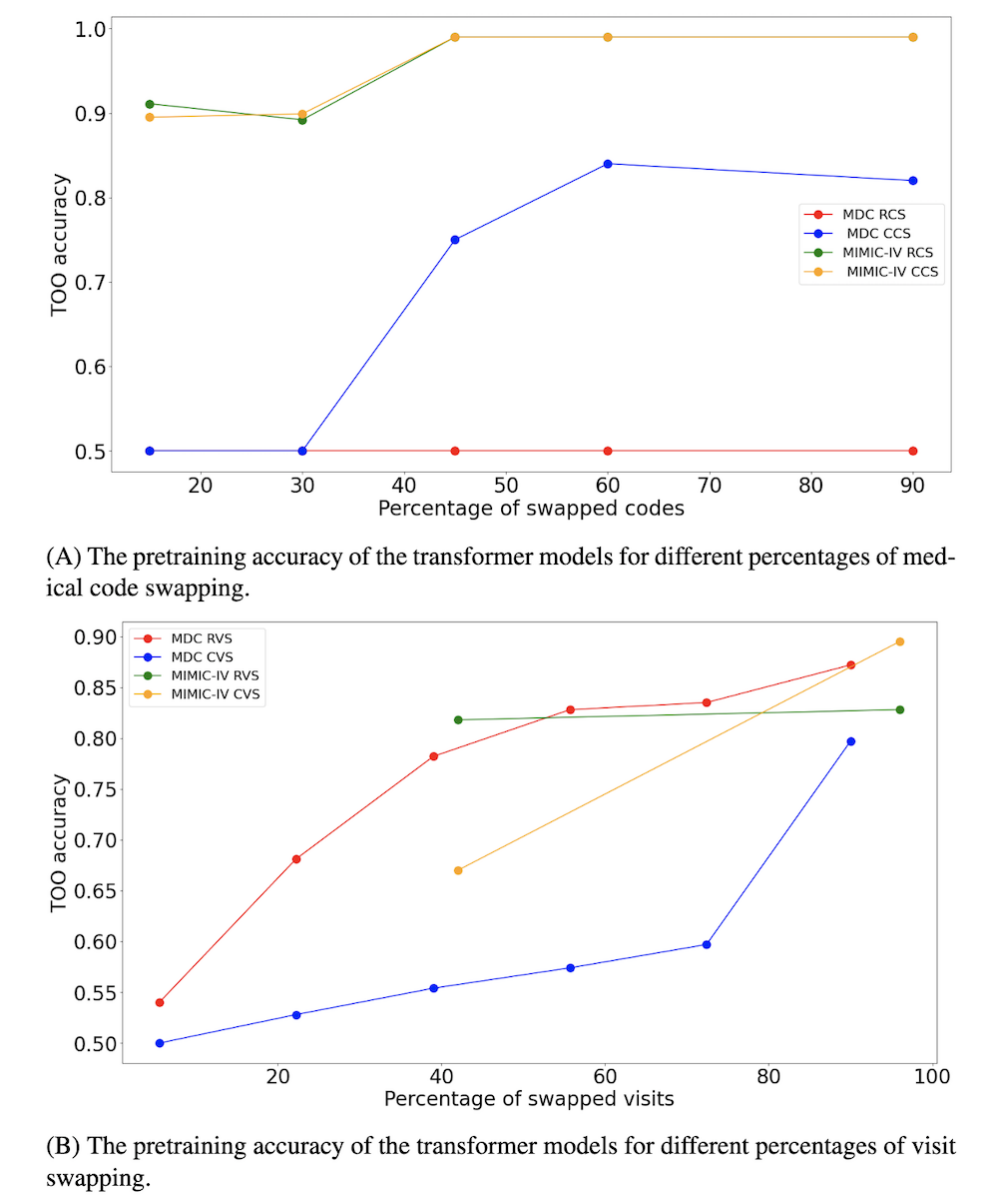
The accuracy of the transformer model in classifying various types of swapping during the pretraining phase on the 10% unseen data from the pretraining split is shown for MDC and MIMIC-IV datasets. (A) The pretrained model can classify permuted samples with even a very low percentage of swapping on the MIMIC-IV dataset. On the other hand, classifying the permuted samples on the MDC was quite challenging. (B) The classification accuracy of the visits-swapped samples increases by raising the number of swapped visits for both methods and both datasets. MDC: Malmo Diet and Cancer Cohort; MIMIC-IV: Medical Information Mart for Intensive Care IV.

We assessed the effectiveness of the transformer models in learning the proposed TOO auxiliary objectives through a series of experiments conducted on the MDC and MIMIC-IV datasets. The four proposed swapping methods (RCS, CCS, RVS, and CVS) were applied with different amounts of swapped code or visit pairs, and the models’ performance in detecting whether a trajectory contained swapped codes or visits was evaluated under varying amounts of swapping. Figure 4 illustrates the impact of increasing the percentage of swaps on classification performance for each dataset and swapping method.

As shown in Figure 4A, for the MIMIC-IV dataset, the transformer model could easily detect swapped trajectories using RCS and CCS methods for swap percentages above 40% (25/62). In contrast, the MDC dataset presented a different challenge. MDC trajectories are, on average, approximately 8 times longer (in terms of visits per patient) than those in MIMIC-IV. Here, the model struggled with the RCS, failing to find solutions effectively across any percentage of swapped pairs. In the CCS task, the model began to classify swapped trajectories more accurately only when the percentage of swaps exceeded 45% (102/254).

The results for the visit-swapping tasks (RVS and CVS) are presented in Figure 4B. These tasks proved more challenging for the models, especially at lower percentages of swaps. For the CVS task in the MDC dataset, the model required over 80% (14/18) of visits to be swapped before achieving satisfactory classification performance. Conversely, in the RVS task, the model achieved 0.8 accuracy with just 40% (7/18) of swapped visits.

For the MIMIC-IV dataset, the CVS task remained difficult for the transformer models, with only a single visit swap (1/2, 42%). However, in the RVS task, the model successfully classified trajectories with only one visit swap, demonstrating greater ease in learning visit-level temporal disruptions in this dataset.

To improve the pretraining performance on the MDC cohort with the RCS swapping method, we used a transfer learning approach. Initial weights from the transformer using the CCS method were used for the RCS task with 45% (102/254) code swapping, and this resulted in an accuracy of 0.684. This approach was also applied during the fine-tuning step for MLM+TOO_RCS_ on the MDC dataset.

### Evaluation of Downstream Tasks

We evaluated the prediction performance of HF and PLS for the MIMIC-IV dataset, while for the MDC dataset, we evaluated the HF and AD prediction performance. The datasets’ specifications and the downstream tasks definitions are described in the section data. The percentages of swapping used for RCS, CCS, RVS, and CVS during pretraining are shown in Multimedia Appendix 1, Table S2 in Multimedia Appendix 1, and the selection was based on the performance on the validation performance within the fine-tuning dataset.

Table 3 shows the performance of Logistic Regression, Random Forest, MLP, Bi-GRU, the transformer pretrained with the MLM auxiliary task, and the 4 variations of the MLM+TOO auxiliary tasks. Consistent with the results of the Med-BERT model [11], the transformer pretrained with only MLM outperformed almost all other conventional methods in all downstream tasks for both datasets.

**Table 3:** Average AUC^a^ values (%) and SD for different methods for the HF^b^ prediction, AD^c^ prediction, and PLS^d^ prediction downstream tasks on the test datasets.

Model or dataset	HF prediction (MDC^e^)	AD prediction (MDC)	HF prediction (MIMIC-IV^f^)	PLS prediction (MIMIC-IV)
Logistic regression	62.4 (1.1)	56.4 (1.1)	83.8 (1.1)	54.2 (0.4)
Random forest	60.7 (0.5)	51.8 (0.3)	78.6 (1.6)	51.1 (0.3)
MLP	67.9 (3.0)	68.0 (1.5)	86.0 (0.5)	59.3 (1.9)
Bi-GRU	62.3 (1.2)	60.4 (1.1)	85.0 (1.3)	55.9 (1.0)
MLM^g^	67.7 (2.6)	69.5 (1.6)	86.2 (0.9)	60.2 (1.2)
MLM+TOO^h^_RCS_^i^	65.1 (1.2)	65.6 (0.7)	88.1 (0.7)	58.4 (0.9)
MLM+TOO_CCS_^j^	64.6 (2.7)	67.2 (1.3)	89.8 (0.8)	60.4 (1.2)
MLM+TOO_RVS_^k^	72.8 (3.1)	70.4 (0.2)	87.9 (1.7)	57.3 (0.8)
MLM+TOO_CVS_^l^	73.9 (1.9)	71.9 (1.6)	87.2 (1.8)	58.8 (1.6)

^a^AUC: area under the receiver operating characteristic curve.

^b^HF: heart failure.

^c^AD: Alzheimer disease.

^d^PLS: prolonged length of stay.

^e^MDC: Malmo Diet and Cancer Cohort.

^f^MIMIC-IV: Medical Information Mart for Intensive Care IV.

^f^gLM: masked language modelling.

^h^TOO: trajectory-order objective.

^i^RCS: random code swapping.

^j^CCS: code swapping function.

^j^RVS: random visit swapping.

^l^CVS: conditional visit swapping.

The visit-level TOO objective methods yielded the best HF and AD prediction performance on the MDC dataset, which features much longer patient trajectories. Using the CVS for the TOO objective achieved the highest area under the receiver operating characteristic curve (AUC) of 0.739 and 0.719 for HF and AD prediction, respectively. Moreover, since the model pretrained with random code swapping exhibited weak performance on the MDC dataset (Figure 4A), we initialized the model with the weights of the CCS pretrained model. We achieved approximately 0.74 accuracy on the TOO objective and subsequently fine-tuned this model for HF prediction, increasing the AUC to 0.65.9. However, this result remained lower than that of the transformer pretrained solely on MLM.

For HF prediction on the MIMIC-IV dataset, the TOO auxiliary task with code swapping improved performance more than other methods. Applying the CCS over the TOO objective achieved the best AUC of 0.89.8. Although predicting the PLS on the next visit is challenging for all models, adding the CCS objective led to the best AUC.

### Performance Boost on Data Insufficiency

**Figure 5:**
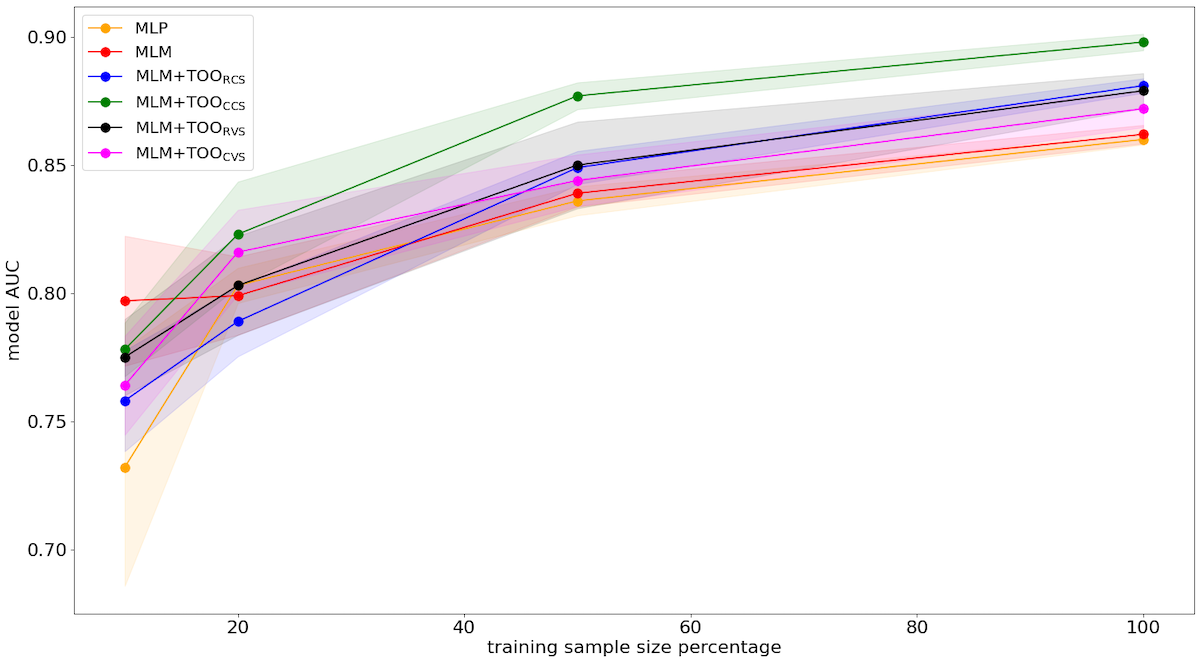
Comparison of HF prediction AUC values for the test sets by fine-tuning on different data sizes on the MIMIC-IV dataset. The shadows represent the 90% CI. AUC: area under the receiver operating characteristic curve; HF: heart failure; MIMIC-IV: Medical Information Mart for Intensive Care IV. MLM: masked language modeling; MLP: multilayer perceptron; TOO: trajectory-order objective.

We further evaluated the impact of combining the proposed TOO with the MLM, using varying fine-tuning sample sizes for predicting HF on the MIMIC-IV test dataset, and compared its performance to the transformer pretrained on MLM and MLP, the most successful conventional method. Fine-tuning sample sizes was reduced to 50% (442/884), 20% (176/884), and 10% (88/884). Figure 5 presents the performance of the MLP (orange line), the transformer pretrained with only MLM (red line), and transformers pretrained with MLM combined with RCS (blue line), CCS (green line), RVS (black line), and CVS (pink line). As the sample size decreased, the transformer pretrained with MLM+TOO_CCS_ achieved higher AUC scores, highlighting its effectiveness in handling data insufficiency.

### The Effect of TOO on Attention Weights

**Figure 6:**
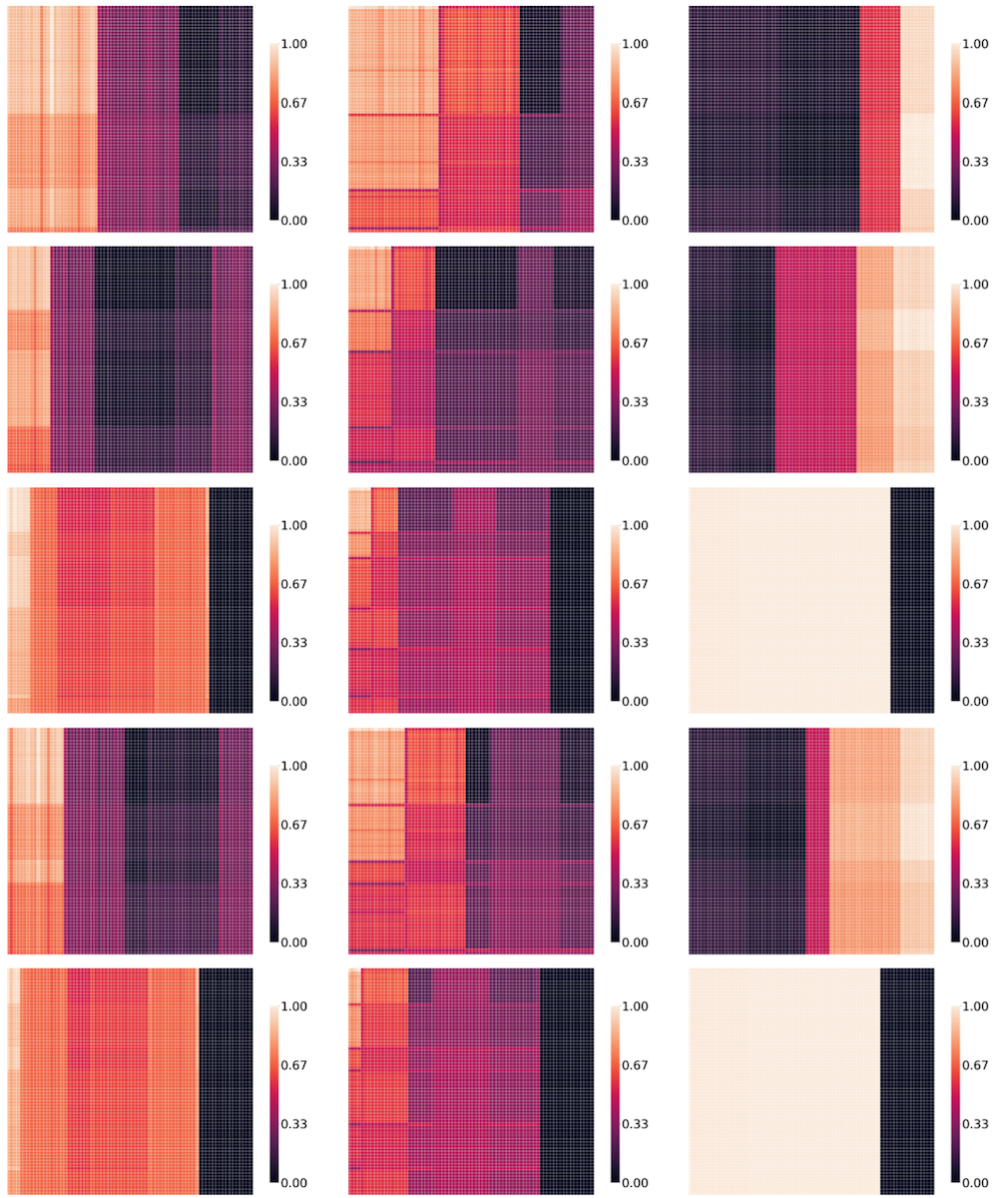
The attention scores (5 heads) for 3 fine-tuned models on HF prediction for the MIMIC-IV dataset, shown for a specific sample from the test set. HF: heart failure; MIMIC-IV: Medical Information Mart for Intensive Care IV.

Visualizing the attention scores, A_h_, of the transformer provides valuable insights into the model’s decision-making process and its representation learning capabilities. More capable models can learn and attend to more complex patterns. Figure 6 shows attention scores for the fine-tuned models based on only MLM, MLM+TOO_RVS_, and MLM+TOO_CCS_ pretraining. The attention scores come from a single patient trajectory for the HF prediction task on the MIMIC-IV dataset. Attention scores for all TOO-BERT variants can be found in Multimedia Appendix 1. In the rightmost column, the attention scores of the model pretrained solely with MLM show a primary focus on the latest codes in the trajectory. In contrast, models pretrained with the TOO objective (shown in the first and second columns) exhibit more diverse attention patterns, capturing more complex and structured relationships across the trajectory. In addition, models pretrained with the visit-level TOO objective (RVS) demonstrated an increased focus on the interactions between sets of consecutive codes (ie, segment-level attention). In contrast, the model pretrained with the CCS objective tended to exhibit attention at the individual code level.

In Figure 6, lighter colors in the heatmap indicate higher attention weights, while darker colors represent lower attention weights. The variations within each model’s attention heads are meaningful, reflecting how the model allocates attention to specific medical codes within the trajectory. The attention scores of the model pretrained on MLM+CCS demonstrate a greater ability to learn complex patterns. For better interpretability, the attention scores for each head are normalized between 0 and 1, though the original values range from 0 to 0.033, with slight variations across different models.

## Discussion

### Principal Findings

The sequential order of the medical codes and their interactions within a patient’s EHR trajectory is crucial for understanding and modeling their health status. While BERT-inspired methods have shown good results in this domain, the challenge lies in capturing the intricate relationships between diseases and prescribed interventions. TOO-BERT enhances the performance of BERT-inspired models by simultaneously learning the order of medical codes and the context of the EHR trajectory. Our approach involves pretraining these models explicitly on the sequential information within a patient’s EHR trajectory, aiming to enhance their learning capability by using the temporal structure. In addition, the introduction of 2 novel weighting methods, CCS and CVS, within the TOO objective enables the models to learn more relevant and frequent causal and correlation dependencies with a data-driven approach.

The pretraining results highlight the differences in learning sequential information between the MIMIC-IV and MDC datasets. The MDC dataset, characterized by an average of approximately 18 visits per patient, presented more challenges in learning single code level sequential information compared to the MIMIC-IV dataset, which has an average of about 2.5 visits per patient (Figure 4A). This discrepancy could stem from the tendencies of transformers to learn the global dependencies and might require additional strategies to capture local patterns as well [50-53]. In addition, the performance of the model initialized with weights from the CCS task in the MDC dataset on the RCS task demonstrated that the proposed conditional probability approach can effectively help the model converge (section Evaluation of pretraining on the TOO auxiliary task).

Combining the proposed TOO with the MLM improved estimated AUC values for all downstream tasks on both datasets. Interestingly, in longer trajectories, visit-level swapping seemed more informative than code-level swapping, suggesting that the TOO auxiliary pretraining objective may improve the efficacy of the transformer in modeling long EHR trajectory data, in addition to suggested architectural improvements [54,55]. Moreover, code-level TOO-BERT reduced the performance of MLM in the MDC dataset, possibly due to the increase in MLM loss associated with adding the TOO auxiliary task during pretraining. Transformers trained solely on MLM demonstrated similar performance to MLP, indicating the complexity of EHR trajectories and data insufficiency in pretraining these models. The addition of the TOO task leveraged the MLM, potentially compensating for data insufficiency in complex models.

Predicting PLS from previous visits based on diagnoses and medications proved to be a particularly challenging task for all models. In addition, while previous research has indicated that BERT-inspired models are excellent few-shot learners [11,24,56], the addition of the TOO auxiliary task showed superior performance with reduced fine-tuning sample sizes for HF prediction in the MIMIC-IV dataset.

The CCS and CVS weighting function enhances the learning process by prioritizing more frequent transitional patterns. This prioritization helps the model focus on meaningful dependencies, such as transitions where the occurrence of one event strongly predicts the occurrence of another. By emphasizing these strong correlations, the model can converge more efficiently and avoid learning from rare or noisy transitions that may not represent meaningful relationships.

This approach is particularly beneficial in the context of small datasets. CCS and CVS ensure the model concentrates on the most informative patterns early in training, which not only facilitates convergence but also helps reduce overfitting to spurious relationships. While larger datasets and highly expressive models may eventually learn such relationships without CCS, the function remains valuable for guiding the model toward robustness in more resource-constrained settings. The other way to conceptualize CCS and CVS is the resemblance to first-order and higher-order Markov chains. CCS amplifies the probability weight of swapping a code pair based on the observation that the occurrence of one code increases the probability of observing the other in future visits. Similarly, CVS approximates a higher-order Markov chain by considering a set of conditions [57].

An interesting finding in our study was that training transformers on sequential information enables them to learn more intricate structures. The variability in the size and number of tiles in the attention weights (Figure 6) suggests that the TOO-objective enabled the transformers to learn a wide range of patterns. However, a quantitative analysis approach would be more suitable for gaining a more concrete understanding of the attention behaviors.

### Limitations

This study has several limitations. The TOO task only considers the order of the medical codes and skips the time irregularity of visits in the EHR [58-60]. Extending the investigation of the new TOO-BERT variant to other datasets with larger sample sizes and longer visit trajectories would enhance our understanding of the differences between code and visit-level swapping pretraining objectives. Furthermore, the inclusion of additional EHR data sources with various modalities, such as test result values and demographic information, with continuous and categorical data types, in TOO-BERT requires further exploration.

### Future Directions

Future research directions may involve a more comprehensive investigation of the challenges associated with the lack of locality in transformers and the exploration of more sample-efficient techniques to enhance the performance of TOO-BERT methods in data-limited scenarios. Enhanced positional encoding techniques and transformer architectures could prove beneficial. The impact of history length, influenced by code and visit level swapping, could be examined by pretraining TOO-BERT on larger datasets with longer visit histories. Furthermore, assessing the performance of pretrained TOO-BERT on other types of downstream tasks or tasks subject to dataset shifts would be valuable.

### Conclusion

In this study, we explored the potential of incorporating the relative positions of medical codes to improve the learned representation of intricate disease-intervention dependencies, especially in scenarios involving lengthy sequences and limited data. Our introduction of TOO-BERT extends the capabilities of the MLM by focusing on the sequential information within patients’ trajectories at both the single code and visit levels. In addition, to enhance the TOO objective, we introduced condition-based code and swapped self-supervised tasks. The outcomes highlight TOO-BERT’s superior performance in predicting PLS, AD, and HF across different sample sizes. Our analysis of attention weights reveals that the TOO task equips transformers to grasp more intricate structural patterns. Future research might involve exploring more sample-efficient pretraining methods and refining transformer architecture and positional encoding to enhance TOO-BERT’s representation learning capabilities further.

## Appendix

Multimedia Appendix 1 Additional material.

## Abbreviations

AD: Alzheimer disease
ATC: Anatomical Therapeutic Chemical Code
AUC: area under the receiver operating characteristic curve
BERT: Bidirectional Encoder Representations from Transformers
Bi-GRU: Bidirectional Gated Recurrent Unit
BYOL: Bootstrap Your Own Latent
CCS: conditional code swapping
CVS: Conditional Visit Swapping
DL: deep learning
EHR: electronic health record
GAN: generative adversarial network
GRACE: Generative Adversarial Networks Enhanced Pretraining
HF: heart failure
Hi-BEHRT: Hierarchical BEHRT
HIPAA: Health Insurance Portability and Accountability Act
ICD: International Classification of Diseases and Related Health Problems
ICD-9: International Classification of Diseases, Ninth Revision
ICD-10: International Statistical Classification of Diseases, Tenth Revision
MDC: Malmo Diet and Cancer Cohort
MIMIC-IV: Medical Information Mart for Intensive Care IV
MLM: masked language modeling
NDC: National Drug Code
NSP: next-sentence prediction
PHI: protected health information
PLS: prolonged length of stay
RCS: random code swapping
RL: representation learning
RVS: random visit swapping
TOO: trajectory-order objective
TOO-BERT: Trajectory Order Objective Bidirectional Encoder Representations from Transformers

## Data Availability

The MIMIC-IV dataset is publicly available from the PhysioNet repository [62]. The Malmo Diet and Cancer Cohort data that support the findings of this study are not publicly available due to data access restrictions imposed by the Malmo Population-Based Cohorts Joint Database. However, the data are available from the corresponding author upon reasonable request and with permission from the Malmo Population-Based Cohorts Joint Database [63].
