# Supplementary material for: Trajectory-Ordered Objectives for Self-Supervised Representation Learning of Temporal Healthcare Data Using Transformers: Model Development and Evaluation Study
Source: JMIR Med Inform. 2025 Jun 4;13:e68138. doi: 10.2196/68138 (PMC12177421; doi:10.2196/68138)
Supplement: Multimedia Appendix 1 [file medinform_v13i1e68138_app1.pdf]

Supplemental Materials: TOO-BERT: A Trajectory Order Objective Bert for self-supervised representation learning of temporal healthcare data

1. Details of Dataset Characteristics

Figure S1 shows the boxplot (excluding outliers) for the number of visits and medical codes per patient in the MDC and MIMIC-IV datasets.

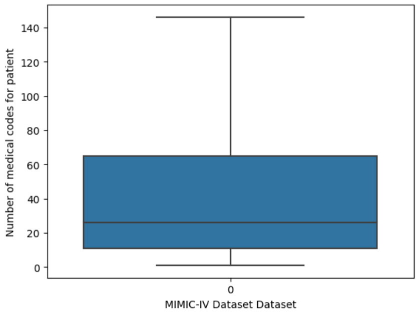

(a) Number of medical codes per patient

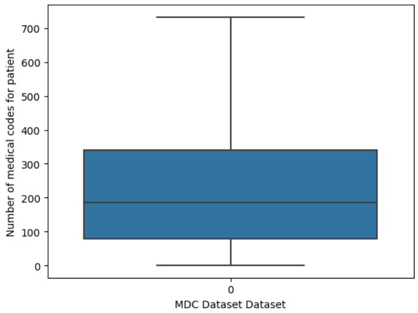

(b) Number of visits per patient

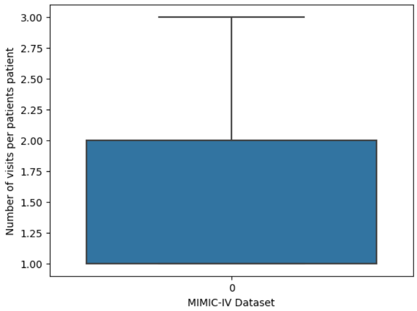

(c) Number of medical codes per patient

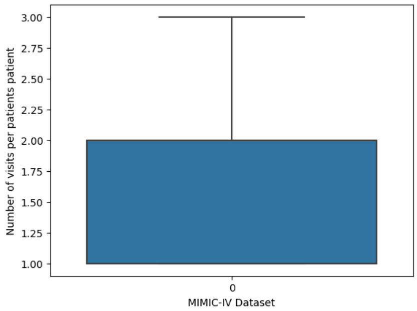

(d) Number of visits per patient

Figure S1. Box plot showing the distribution of the number of medical codes and visits per patient in the MIMIC-IV and MDC datasets.

2. Exclusion of Less Frequent Medical Codes

We set a minimum frequency threshold of 20 for both datasets. This threshold was selected as it approximately corresponds to the median (50th quantile) of the frequency distribution of unique diagnoses in both datasets. Codes with a frequency below this threshold were excluded, as they appeared too infrequently to provide meaningful learning signals for the model.

The initial and final numbers of unique codes after applying the frequency threshold are as follows:

2.1. MDC Dataset

- **Before excluding less frequent codes:**
  * Number of unique diagnoses: 3,782
  * Number of unique medications: 140
- **After excluding codes with a minimum frequency of 20:**
  * Number of unique diagnoses: 1,558
  * Number of unique medications: 111

2.2. MIMIC-IV Dataset

- **Before excluding less frequent codes:**
  * Number of unique diagnoses: 4,530
  * Number of unique medications: 157
- **After excluding codes with a minimum frequency of 20:**
  * Number of unique diagnoses: 2,195
  * Number of unique medications: 137

3. Percentages of Swapped Codes/Visits

We fine-tuned the swapping percentage for the four variations of MLM+TOO models, selecting the values that yielded the best performance for the HF prediction on the fine-tuning split for each dataset. Table 1 shows the percentages of code/visit swaps used for each dataset.

Table 1. Percentages of swapping used for different models on the MDC and MIMIC-IV datasets.

Swapping Method / Dataset	MDC	MIMIC-IV
RCS	90%	30%
CCS	45%	30%
RVS	39%	99%
CVS	90%	99%

4. Distribution of Positive Cases Across Downstream Tasks

Table 2 summarizes the percentage of positive cases across the different downstream tasks.

Table 2. Percentage of Positive Cases in Different Downstream Tasks

**Task**	**% Positive Labels**
PLS	28
HF (MIMIC-IV)	27
AD	9
HF (MDC)	25

5. Size of the Sliding Window for Making Trajectories in the Pre-training Phase

During the pre-training phase, we utilized the 0.7 quantile of the trajectory lengths in each dataset to determine the fixed sliding window size. This approach ensures that the window size is representative of the distribution of trajectory lengths in the datasets:

- For the **MDC dataset**, the sliding window size is **131**.
- For the **MIMIC-IV dataset**, the sliding window size is **65**.

6. Scaling in CCS weighting function

We used the following function for scaling the CCS:

$$S_{i,j} = \begin{cases} \alpha \cdot R_{D2M}, & \text{if } i \in \{\, diagnoses\}, j \in \{\, medications\} \\ \alpha \cdot R_{D2M}, & \text{if } i \in \{\, medications\}, j \in \{\, diagnoses\} \\ \alpha^2 \cdot R_{D2M}, & \text{if } i \in \{\, diagnoses\}, j \in \{\, diagnosis\} \\ 1, & \text{if } i \in \{\, medications\}, j \in \{\, medications\} \end{cases} \tag{1}$$

Where $R_{D2M} = \frac{\text{\#unique diagnosis}}{\text{\#unique medications}}$ is the ratio between the number of unique medications and unique diagnoses, and α is an arbitrary coefficient (we used $\alpha = 0.5$). The scaling function is designed to address the sparsity in diagnosis codes compared to medication codes, with diagnosis codes being approximately eight times less frequent. Moreover, to make the weighting matrix smoother and more resilient to small changes, we aggregated weights for medical codes sharing the same first three digits by summation.

7. Selection of the Transformer Head Dimension

The head dimension of the transformer was selected based on empirical evaluations performed during our experiments. We tested transformer models with head dimensions of 16, 36, and 64 to predict heart failure. The resulting AUC scores were as follows:

- **Head dimension = 16:** AUC = 86.7
- **Head dimension = 36:** AUC = 89.3
- **Head dimension = 64:** AUC = 88.9

From these results, it is evident that a head dimension of 36 provided the best performance, achieving the highest AUC score of 89.3. Increasing the head dimension to 64 did not improve performance further and resulted in significantly higher computational cost and memory usage during training. Conversely, a head dimension of 16 led to a decrease in performance, likely due to insufficient representation capacity. Based on this analysis, we determined that a head dimension of 36 offers the optimal trade-off between model performance and computational efficiency for our specific task and datasets.

8. The effect of TOO on attention weights

Figure S2 represents the fine-tuned pretrained models with $MLM + RVS$, $MLM + CVS$, $MLM + RCS$, $MLM + CCS$ and $MLMonly$ for the HF prediction task on the MIMIC-IV dataset. We examined the attention score for a single patient trajectory. The lighter colors in the heatmap indicate higher attention weights, while darker colors represent lower attention weights. Each attention head operates on its own scale; therefore, attention scores are not directly comparable across different heads. However, the variations within each attention head are meaningful, as they represent how the model allocates attention to specific medical codes within the trajectory. Models pre-trained with the TOO objective demonstrate more diverse and structured attention patterns. These patterns enable the model to capture more complex relationships among medical codes and their temporal progression.

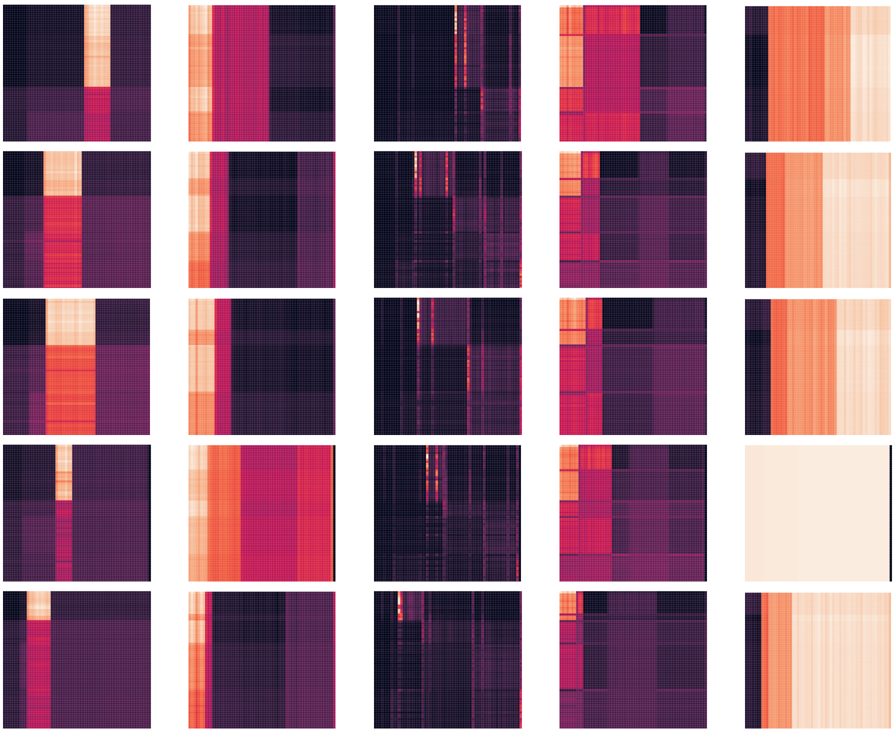

(a) MLM + RVS

(b) MLM + CVS

(c) MLM + RCS

(d) MLM + CCS

(e) MLM only

Figure S2. The attention scores (five heads) for five fine-tuned models on HF prediction for the MIMIC-IV dataset, shown for a specific sample from the test set. The attention scores of the model pre-trained on $MLM + CCS$ demonstrate a greater ability to learn complex patterns.
